# Constraint-induced movement therapy reduced shoulder pain and improved function in subacute and chronic stroke: a cohort study

**DOI:** 10.3389/fneur.2025.1639840

**Published:** 2025-09-04

**Authors:** Annika Sefastsson, Therése Brändström, Håkan Littbrand, Per Wester, Ann Sörlin, Britt-Marie Stålnacke, Per Liv, Xiaolei Hu

**Affiliations:** ^1^Department of Community Medicine and Rehabilitation, Rehabilitation Medicine, Umeå University, Umeå, Sweden; ^2^Liljeholmskliniken, Stockholm, Sweden; ^3^Department of Community and Rehabilitation Medicine, Geriatric Medicine, Umeå University, Umeå, Sweden; ^4^Department of Public Health and Clinical Medicine, Umeå University, Umeå, Sweden; ^5^Department of Clinical Science, Karolinska Institute Danderyd Hospital, Stockholm, Sweden

**Keywords:** constraint-induced movement therapy, shoulder pain, shoulder range of motion, stroke rehabilitation, upper extremity motor function, range of motion (ROM), motor function

## Abstract

**Introduction:**

The objective of this study was to evaluate the effects of Constraint-Induced Movement Therapy (CIMT) on hemiplegic shoulder pain (HSP), shoulder range of motion (ROM) and upper extremity motor function in stroke patients.

**Methods:**

This longitudinal intervention cohort study was performed in an outpatient clinic without a control group. Participants underwent individually tailored CIMT with a patient therapist ratio of 4:1 for 6 h/day, 5 days/week for 2 consecutive weeks, including daily shoulder strength and joint motion training. A total of 221 (101 with and 120 without pre-CIMT HSP) middle-aged (median 54 years) persons at sub-acute or chronic phases after stroke were included in the study. The Fugl-Meyer Assessment (FMA) subscale for pain was used for defining and scoring HSP at passive motion (sum of four directions of movement, maximum 8 points indicating no pain). Passive and active shoulder ROM (sum of flexion and abduction) were assessed. Upper extremity motor function was assessed with B. Lindmark Motor Assessment. Assessments were done pre- and post-CIMT and at 3-month follow-up. Comparisons were stratified by subgroups with- and without HSP.

**Results:**

In the subgroup with pre-CIMT HSP, median HSP score at passive movement was reduced (FMA shoulder pain score increased) from pre- to post-CIMT from 5 points to 7 points post-CIMT, (*p* < 0.001, Effect size (ES) 0.68). Median active ROM increased from 230° to 308° (*p* < 0.001, ES 0.72) and median passive ROM increased from 350° to 360° (*p* < 0.001, ES 0.44). Median motor function improved from 42 to 49 points (*p* < 0.001, ES 0.92). In the subgroup without pre-CIMT HSP no statistically significant increase of HSP was seen and no clinically significant changes observed for active or passive ROM after CIMT. Median motor function improved from 52 to 56 points (*p* < 0.001, ES 0.71). All improvements persisted at 3-month follow-up.

**Conclusion:**

CIMT in an outpatient clinical setting may be a feasible treatment to decrease HSP and to improve shoulder ROM and upper extremity motor function among middle-aged persons in the subacute and chronic phases after stroke. Results need to be confirmed in an RCT setting.

## 1 Introduction

Hemiplegic shoulder pain (HSP) is a common post stroke complication, which may negatively impact the stroke patient’s ability to respond to rehabilitation and consequently, to reach an optimal level of function and activity (1). A recent systematic review identified poor arm motor function and reduced passive shoulder range of motion (ROM) for flexion and abduction to be risk factors for developing HSP (1). An observational study reported the prevalence of HSP to be 55% at 4 months post stroke (2) and a systematic review reported the prevalence to be 39% at 12 months (3).

Despite extensive research, causes of HSP are not fully understood, and consensus regarding etiology and effective treatment approaches has not been reached (3–6). Further research is needed since results regarding effective treatment are inconclusive and sometimes even contradictory (7, 8). HSP in the subacute and chronic phases is considered under-diagnosed (9, 10) and only a smaller portion of HSP patients receive pain treatment in the later phases (11, 12) indicating that HSP may also be an under-treated diagnosis (10). Several pharmacological and non-pharmacological treatment interventions have demonstrated different degrees of effectiveness on HSP (4, 7, 13–15).

The original Constraint-Induced Movement Therapy (CIMT) and different versions of modified CIMT protocols have demonstrated to be effective for improving upper limb motor function post stroke with a good carry-over effect to real world situations (16–18). CIMT and/or modified CIMT are recommended in the Swedish (19) as well as in international clinical guidelines (20). The aspect of whether the highly intensive CIMT protocol might worsen shoulder pain has been addressed with the conclusion that CIMT did not affect shoulder pain negatively (21). However, a possible positive effect of CIMT on shoulder pain has, to the best of our knowledge, not yet been addressed.

The objective of this study was to investigate the effects of CIMT, administered in an outpatient clinical setting, on HSP, passive and active shoulder ROM and upper extremity motor function in the subacute and chronic stages post stroke with stratification of presence or absence of pre-CIMT HSP.

## 2 Materials and methods

### 2.1 Study design

The present intervention study is a longitudinal cohort study without control group, carried out in an outpatient physiotherapy clinic in Stockholm, Sweden. Patients were mostly referred to CIMT from local hospitals after having undergone standard rehabilitation routines.

Data was collected retrospectively from medical records between 2000 and 2014 and thereafter prospectively until 2018, all with an identical study protocol. All participants provided written informed consent prior to study inclusion. The study conformed to the Helsinki declaration. Ethical approval was obtained from the regional Ethical Review Board in Umeå and, as of 2019, The Swedish Ethical Review Authority, Sweden (Dnr. 2013–327-31 M).

### 2.2 Participants

Participants were recruited from the outpatient physiotherapy clinic, where CIMT was offered to patients meeting the following criteria: motivation to participate, minimum 1 month since acquired brain injury, ability to understand instructions, ability to walk indoors without an assistive device or using a wheelchair, ability to repeat minimum wrist extension 20 degrees and finger extension 10 degrees. Patients with unstable medical conditions, as assessed by the physician, were not referred to CIMT. Efforts were made to contact all patients who participated in CIMT between 2000 and 2014 to invite them to participate in the study. Regarding the prospective part of the study, patients were invited to participate in the study at their first visit to the clinic. The same protocol was used throughout the study apart from consent being collected after/before CIMT.

Inclusion criteria for the present study, in addition to having participated in CIMT at the above clinic, was written informed consent and first ever CIMT. Exclusion criteria were: brain injury other than stroke, age <18 years, no available data on pre-CIMT HSP (HSP defined as reduced Fugl-Meyer Assessment (FMA) score for shoulder pain).

### 2.3 Intervention

Treatment followed the main principles of the original CIMT protocol (22, 23) with the exceptions of treatment being carried out in groups of four participants and one physiotherapist (PT) (the first author) whereas the original concept was one participant to one therapist. We also put enhanced emphasis on strength and joint mobility exercises. Since modified CIMT, according to consensus, entails specific modifications regarding reduced daily treatment time, use of constraint and transfer package we chose to label our intervention CIMT despite some modifications (23).

#### 2.3.1 Intensive supervised training

The intervention encompassed intensive massed practice 6 h/day (8 × 45 min/day) for 10 consecutive weekdays with a patient therapist ratio of 4:1. Participants had an individually tailored training program, based on pretreatment assessments and the patient’s stipulated goals. A daily revision of the training programs was done to ensure that exercises were optimally challenging. Lunch was considered part of the treatment since eating and drinking was done with the weaker hand. Utmost care was taken to avoid compensatory movements when performing motor- and activity enhancing exercises.

Daily strength and joint motion exercises were performed with 45 min of focus on each; shoulder, arm/wrist and hand/fingers. For the HSP group, strength exercises were performed even when initially provoking pain. Any painful exercise was discontinued if causing increased pain after several repetitions but then tried again with adequate alteration to prevent further pain. Participants were given personalized home exercises to do on weekends.

#### 2.3.2 Motor training based on shaping strategy

The shaping strategy encompassed more complex activities being practiced in small steps with a gradually increasing level of difficulty to ensure that goals were achievable. The achieved skills were eventually assembled into a more complete activity, which could be expressed as “practicing parts” before “practicing tasks.”

#### 2.3.3 Constraining mitt on the affected hand for 90% of waking hours

Participants consented to wearing a padded mitt on the less affected hand for at least 90% of waking hours during the two-week intervention. A written agreement was signed, stating situations constituting exceptions from the agreement specified in detail.

#### 2.3.4 Transfer package

A series of behavioral aspects were implicated. There was a daily informal follow-up on how much and how well the weaker hand had been used during time away from the clinic.

After completing CIMT, participants received a training program which they were recommended to do 4–7 days/week until the 3-month follow-up. The content and frequency with which to perform the program was decided in collaboration with the individual to enhance the feeling of responsibility and commitment. Participants were encouraged to keep a simple diary of the extent of training performed. There was merely an oral inquiry on compliance with the home exercise program at follow-up.

### 2.4 Outcomes

Assessments were administered pre- and post-CIMT and at 3-month follow-up and were performed by one physiotherapist (the first author). Test results from previous assessments were not available at retest. Tests were performed according to a written protocol to ensure standardized administration.

#### 2.4.1 Shoulder pain

Shoulder pain during passive flexion, abduction, external and internal rotation was assessed using the FMA score. The FMA was developed to evaluate recovery from hemiplegic stroke (24). The assessment records pain on a 3-point ordinal scale 0–2, where 2 indicates no pain, 1 some pain and 0 pronounced pain and followingly a total FMA shoulder pain score of 8 points indicates no pain. Having no pre-CIMT HSP was defined as having 2 points on each of the four tested directions of motion (a total of 8 points for all four directions). Pre-CIMT HSP was defined as having 0 or 1 in any or all of the four directions of motion. Maximum pain intensity was recorded as the highest pain intensity (the lowest FMA score) reported in any of the four directions of movement.

#### 2.4.2 Shoulder range of motion

Passive and active ROM were assessed using goniometry (25) and recorded as the sum of ROM for flexion and abduction (normal range 0–180° respectively). Tests were performed in a sitting position.

#### 2.4.3 Motor function

The full version of the B Lindmark Motor Assessment (BLMA) (26) was used to evaluate motor function. The assessment is based on the FMA and evaluates bilaterally on a 4-point scale, from 0 to 3, where 3 is normal or almost normal function and 0 indicates no function. Compensatory use of involved joints for any of the items renders reduced scoring. In this study we used subscale A, investigating upper extremity motor function with 19 items and a maximum total score of 57 points. The assessment has proven to be valid and reliable (27).

### 2.5 Data presentations and statistical analysis

Descriptive statistics for baseline characteristics, including motor function and active/passive ROM, were summarized and are reported as frequencies and percentages or as medians and interquartile ranges (IQR) both for the cohort as a whole and for subgroups with and without HSP. Drop-out analyses comparing baseline characteristics between the drop-out group and follow-up group were also performed using the Chi-square or Mann–Whitney U-tests.

Maximum pain intensity was reported as percentages, which were calculated as the number of individuals in the pain category (no pain, some pain or pronounced pain) divided by the total number of individuals in the group at each time-point.

All other outcome variables are presented as medians and interquartile ranges (IQRs). Outcome data were stratified and analyzed separately for participants with and without HSP.

Due to data skewness and ceiling effects, as well as FMA shoulder pain score and BLMA being based on ordinal scales, the within-group pairwise comparisons of outcome variables between different time points were performed using the Sign Test. Comparing the subgroups with and without HSP was not considered relevant due to ceiling effects in the no-HSP group. *p*-values were adjusted for repeated comparisons between time-points (i.e., pre-CIMT to post-CIMT, pre-CIMT to follow-up and post-CIMT to follow-up) using Bonferroni correction. Effect sizes (ES) were calculated as Probability of Superiority for dependent samples, PS*_dep_*, defined as the number of positive differences divided by the total number of pairs (n_positive differences_/N_pairs_). This measure essentially reflects the proportion of participants who improved, thus making interpretation intuitive.

Data were analyzed using the IBM SPSS Statistics software, version 29.0.1.0. Figures were generated using the GraphPad Prism 10, version 10.3.0. A two-tailed *p-*value <0.05 was considered significant.

## 3 Results

**Figure 1:**
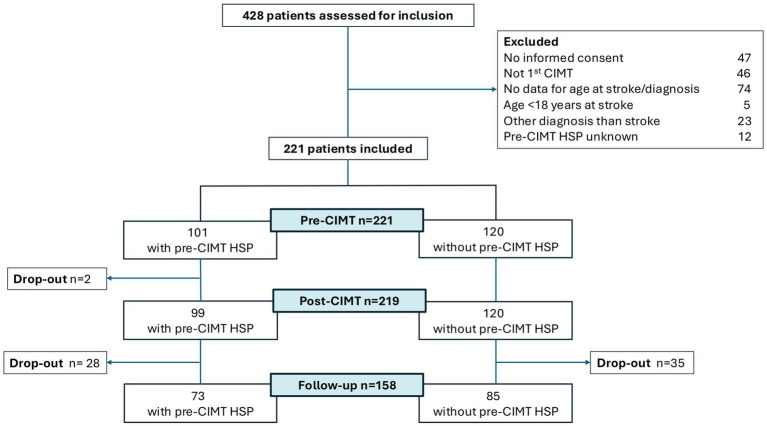
Flowchart of participant recruitment and drop-out.

Out of 428 patients who participated in CIMT at the clinic and were assessed for study eligibility 221 subjects were included in the study after application of inclusion and exclusion criteria, 101 (46%) with pre-CIMT shoulder pain (HSP group) and 120 without pre-CIMT shoulder pain (no HSP group). There was a 28.5% drop out rate at follow-up consisting of 28 participants with and 35 without shoulder pain (Figure 1). Drop-out analysis showed that the drop-out group did not differ from the follow-up group regarding any of the characteristics presented in Table 1, such as age (*p* = 0.554), gender (*p* = 0.226), affected side (*p* = 0.624), motor function (*p* = 0.983) or prevalence of pre-CIMT shoulder pain (*p* = 0.813).

**Table 1:** Descriptive characteristic at baseline for the total cohort and participants divided into subgroups with and without hemiplegic shoulder pain (HSP and no HSP).

	Total (*n* = 221)		With pre-CIMT HSP (*n* = 101)		Without pre-CIMT HSP (*n* = 120)	
	*n* (%)	Median (IQR)	*n* (%)	Median (IQR)	*n* (%)	Median (IQR)
Gender (F/M)	84/137 (38/62)		35/66 (34.7/65.3)		49/71 (40.8/59.2)	
Age at stroke (years)		54 (46–61)		58 (51–63)		52 (43–58)
Time from stroke to CIMT (months)		9 (5–14)		9 (5–14)		9 (4–14)
Type of stroke (infarction/hemorrhagic/ not specified)	77/46/98 (34.8/20.8/44.3)		28/20/53 (27.7/19.8/52.5)		49/26/45 (40.8/21.7/37.5)	
Affected side (right/left)	111/110 (50.2/49.8)		51/50 (50.5/49.5)		60/60 (50/50)	
Dominant hand (right/left)	199/21 (90.5/9.5)		89/12 (88.1/11.9)		110/9 (92.4/7.6)	
Dominant hand affected (yes/no)	113/107 (51.4/48.6)		51/50 (50.5/49.5)		62/57 (47.9/52.1)	
Motor function, BLMA, pre-CIMT (points, max 57)		47 (34–54)		42 (32–49)		52 (40–56)
Active ROM, flexion, pre-CIMT (°)		178 (120–180)		130 (80–170)		180 (175–180)
Active ROM, abduction, pre-CIMT (°)		180 (90–180)		95 (70–175)		180 (180–180)
Passive ROM, flexion, pre-CIMT (°)		180 (175–180)		170 (158–180)		180 (180–180)
Passive ROM, abduction, pre-CIMT (°)		180 (180–180)		180 (160–180)		180 (180–180)

### 3.1 Descriptive characteristics

Demographic and baseline characteristics are presented in Table 1. In the present cohort, the median of age was 54 years old with 90.5% right handed. Males represented 62% of participants. The median time from stroke to CIMT was 9 months. In our cohort of 221, 45.7% had pre-CIMT shoulder pain at passive joint motion.

### 3.2 Reduced shoulder pain after CIMT

#### 3.2.1 HSP group

**Figure 2:**
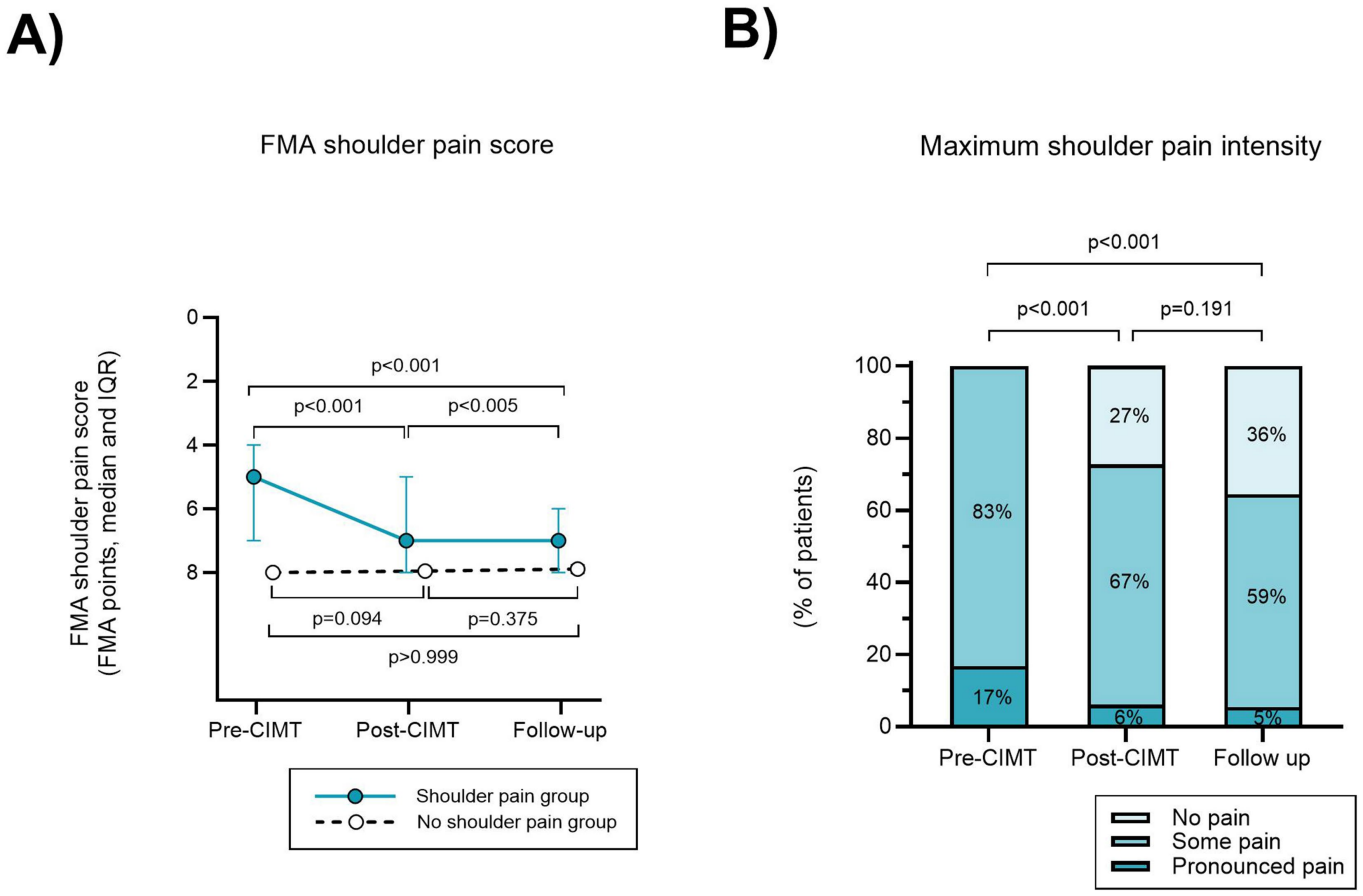
Reduced shoulder pain after CIMT in the group with pre-CIMT hemiplegic shoulder pain. **(A)** The total of FMA shoulder pain scores in four directions of motion (flexion, abduction, external- and internal rotation) are presented on a reversed scale as a higher score indicates less pain. **(B)** In the hemiplegic shoulder pain group, maximum shoulder pain intensity for each time of assessment was scored as the highest degree of shoulder pain in any of the four directions of motion tested.

FMA shoulder pain score improved from pre- to post-CIMT, and from pre-CIMT to follow-up, with a change in median FMA shoulder pain score from 5 (IQR 4–7) to 7 in both comparisons (IQR 5–8 and 6–8) (*p* < 0.001, ES 0.68 and 0.79), Figure 2A. 6 participants had a higher shoulder pain score post-CIMT. The increase in pain post-CIMT did not persist at follow-up in 5 of 6 participants (1 individual had missing data at follow-up). Maximum shoulder pain intensity decreased from pre-CIMT to post-CIMT and from pre-CIMT to follow-up (*p* < 0.001 and ES 0.35 and 0.33), Figure 2B.

#### 3.2.2 No HSP group

At post-CIMT 5.0% of participants in the no HSP group recorded shoulder pain and at follow-up the prevalence of shoulder pain was 4.7%, however these results were not statistically significant (*p* = 0.093 and 0.375), Figure 2A.

### 3.3 Improved active ROM

#### 3.3.1 HSP group

**Figure 3:**
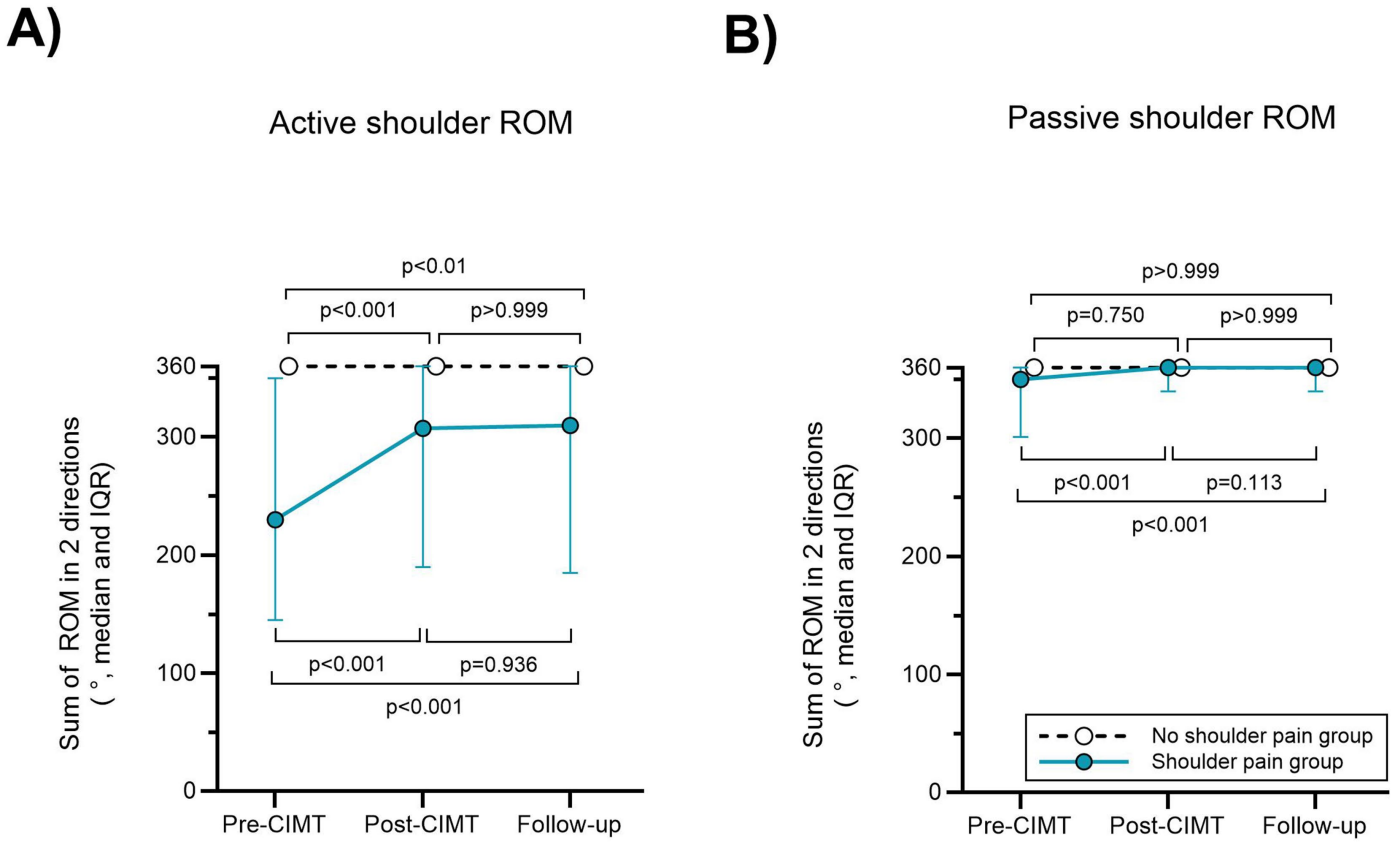
Shoulder ROM after CIMT. **(A)** Active and **(B)** passive ROM for the shoulder pain and no shoulder pain groups. Data are reported as sum of ROM for flexion and abduction.

Active shoulder ROM increased from pre- to post-CIMT [median 230° (IQR 145–350) to 308° (IQR 190–360)] and from pre-CIMT to follow-up [median 230° (IQR 145–350) to 310° (IQR 185–360), *p* < 0.001 for both comparisons, ES 0.72 and 0.73 respectively], Figure 3A.

#### 3.3.2 No HSP group

In the no HSP group, most participants had full active shoulder ROM at pre-CIMT, hence no clinically relevant increase was possible, nevertheless there was a statistically significant difference from pre-to post-CIMT (*p* < 0.001, ES 0.23), and from pre-CIMT to follow-up (*p* < 0.01, ES 0.19), Figure 3A.

### 3.4 Improved passive ROM

#### 3.4.1 HSP group

Improved passive ROM was observed from pre- to post-CIMT and from pre-CIMT to follow-up (median 350° (IQR 303–360) to 360° (IQR 340–360), *p* < 0.001 in both comparisons, ES 0.44 and 0.51), Figure 3B.

#### 3.4.2 No HSP group

In the no HSP group, we did not observe an improvement for a significant number of participants, Figure 3B.

### 3.5 Improved arm and hand motor function

#### 3.5.1 HSP group

**Figure 4:**
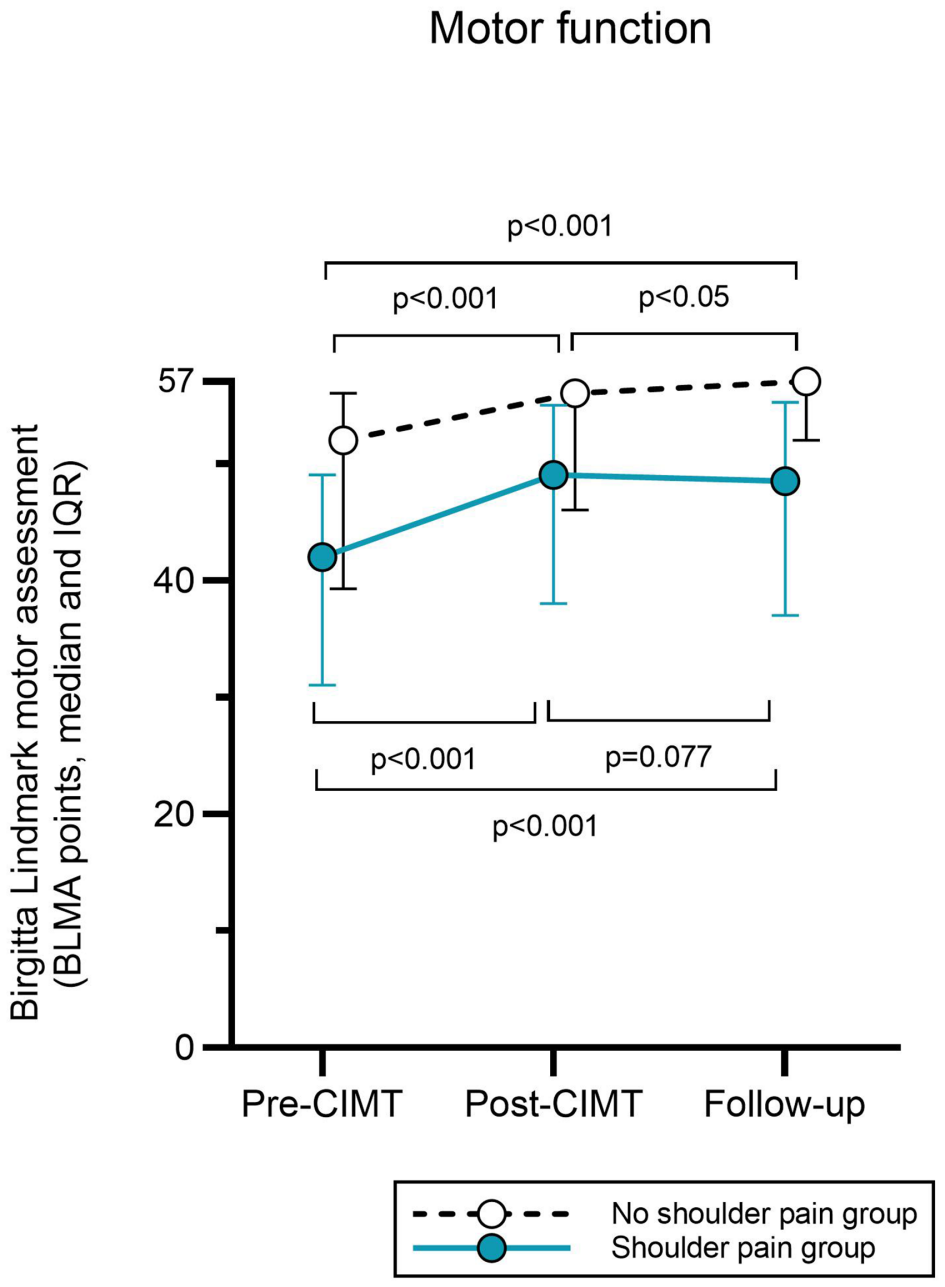
Motor function measured by the B Lindmark Motor Assessment (max 57 points) improved after CIMT in both the group with- and the group without hemiplegic shoulder pain.

The BLMA score increased from pre- to post-CIMT and from pre-CIMT to follow-up (median score 42 (IQR 32–49) to 49 (IQR 38–55) and 48.5 (IQR 37–55), *p* < 0.001 in both comparisons, ES 0.92 and 0.91 respectively), Figure 4.

#### 3.5.2 No HSP group

In the no HSP group BLMA score improved from pre- to post CIMT and from pre-CIMT to follow-up (median score 52 (IQR 40–56) to 56 (IQR 46–57) and 57 (IQR 52–57) points, p < 0.001 for both comparisons, ES 0.71 and ES 0.74), Figure 4.

## 4 Discussion

In this large longitudinal cohort study, we efficiently applied individually tailored CIMT among middle-aged subjects in the subacute and chronic stages post stroke in an outpatient clinical setting. The most striking finding in the current study is that the group with HSP showed reduced pain during passive shoulder movements after 2 weeks of CIMT with effects remaining at 3-month follow-up. Moreover, fully one fourth of participants became pain free after CIMT and at follow-up more than one third were pain free. In addition to the pain-relieving effect at passive movement, CIMT simultaneously improved passive and active shoulder ROM and motor function in the HSP group. The no-HSP group had significantly better passive and active ROM and motor function pre-CIMT but nonetheless improved active shoulder ROM and motor function significantly. Improvements persisted at follow-up for both subgroups.

Although the pain-relieving effect in the current study is exciting and encouraging, we acknowledge that it is a single-center clinical study without a control group. However, our participants performed 2 weeks of high intensity training with a majority of them being in a chronic stage post-stroke, where spontaneous recovery was not to be expected. Thus, we are inclined to argue that the most likely explanation to the sizeable pain-relieving effect in only 2 weeks is the CIMT intervention.

Our findings on CIMT alleviating shoulder pain are exciting and truly novel. To the best of our knowledge no other study has explored this possibility. The EXCITE study reported merely that shoulder pain did not worsen after CIMT in a much smaller cohort (21). RCT studies have tended to exclude patients with HSP (17, 28, 29) from concerns of worsening pain.

Furthermore, our findings may have important clinical implications since current clinical guidelines recommend the use of a supportive and hence immobilizing device for patients with HSP (20). Although the mechanism behind the pain-relieving effect of CIMT on HSP is unclear, our results support active mobilization and strength and joint motion exercises of the shoulder to reduce HSP. This is in line with routines in orthopedic medicine where immobilization of the shoulder joint is avoided even for shorter time periods from knowledge that immobilization may cause pain (30). Interestingly our findings are also in line with results from a recent systematic review on post-stroke neuropathic shoulder pain that recommends strength exercises to reduce pain (29). For most participants in our HSP group the pain is likely to be nociceptive, however research has indicated that approximately 10% of HSP is of neuropathic origin (11, 31). Our results indicate that CIMT may reduce, and even cure, HSP for stroke patients with some as well as pronounced shoulder pain.

In our study, shoulder strength and motion exercises were performed even when initially causing pain. This could be considered controversial and not in line with current consensus in stroke rehabilitation but was based on clinical experience from working with CIMT. A small number of participants in both the HSP and no HSP group experienced exacerbation of shoulder pain post-CIMT. However, in the HSP group the majority improved to follow-up while there was a status quo for the no HSP group. Our results thereby lessen concerns about the risk of CIMT affecting shoulder pain negatively, which is in line with findings in the EXCITE study (21).

In addition to a pain-relieving effect, CIMT improved passive and active shoulder ROM and upper extremity motor function. These results confirm the efficacy of CIMT previously demonstrated in RCT studies (32, 33) and thus provide evidence supporting CIMT to be a feasible treatment intervention also in real-world outpatient clinical settings with less strict inclusion criteria compared to RCT studies. Notably, participants in our study demonstrated good passive ROM with a median value approaching full range pre-CIMT. The CIMT inclusion criteria, excluding participants with severely reduced motor function for wrist and fingers, likely introduced a positive selection bias toward participants with good ROM, explaining part of our findings. Good shoulder ROM may not be uncommon in stroke patients as indicated by a study reporting full or almost full passive shoulder ROM in 46% of participants in late subacute phase post-stroke (34). A good ROM in turn implies a low degree of general spasticity (35). Hence, our findings contrast with previous studies focused on spasticity in individuals with acquired brain injury, where impaired passive ROM has been reported (36, 37).

Improved passive and active ROM are essential for normalization of motor function as opposed to using compensatory strategies. The BLMA rating scale reduces the given score when a task is performed using compensatory strategies, this leads us to suggest that improved motor function on BLMA, as seen in our study, reflects true recovery rather than compensation (23). This idea is contrary to established knowledge on improvements in late stages being based on compensatory strategies (23). We suspect that the improved level of motor function in our study has been used by our participants in everyday life and thus together with the prescribed home exercise program has contributed to results remaining intact at 3-month follow-up. Our results support that CIMT has a good carry-over effect of motor improvements to the real-world setting (16). The HSP group exhibited significantly lower scores for ROM and motor function before CIMT, consistent with the previously known association between HSP, ROM and motor function (1, 2).

Participants in the HSP group benefited considerably from CIMT. This finding is contrary to previous research (38) indicating that HSP negatively impacts the stroke patient’s ability to respond as well to rehabilitation. Despite considerable improvements for the HSP group, they merely reached the level of motor function that no HSP group had pre-CIMT. This raises the question whether the HSP group might have benefited from additional CIMT treatment.

### 4.1 Study strengths and limitations

A strength of our study is a relatively large cohort with 221 participants, including 101 participants with HSP. The lack of a control group is an obvious study limitation. All treatment and tests performed by the same PT renders consistency to the assessment procedure but is simultaneously a risk of bias. Seeking to lessen this risk, previous test results were not available to the PT at subsequent assessment. Drop-out at follow-up is a potential cause of bias, however the drop-out analysis revealed no significant differences between the drop-out and follow-up groups. Inclusion and exclusion criteria demanded cognitive and motor function to be relatively high and the median age was 54 years; factors that limit the generalizability of our results. Mixing retrospective and prospective cohorts may entail a risk of bias.

## 5 Conclusion

In conclusion, individually tailored CIMT may be a feasible and useful treatment, in an outpatient clinical setting, of middle-aged stroke patients, to reduce shoulder pain and improve shoulder ROM and motor function in the subacute and chronic stages. The pain-relieving effect of CIMT on shoulder pain, demonstrated in this uncontrolled study, needs to be evaluated in RCT studies.

## Data Availability

The raw data supporting the conclusions of this article will be made available by the authors, without undue reservation.
